# Recognition of RNA duplexes by chemically modified triplex-forming oligonucleotides

**DOI:** 10.1093/nar/gkt352

**Published:** 2013-05-08

**Authors:** Yuan Zhou, Elzbieta Kierzek, Zi Ping Loo, Meraldo Antonio, Yin Hoe Yau, York Wieo Chuah, Susana Geifman-Shochat, Ryszard Kierzek, Gang Chen

**Affiliations:** ^1^Division of Chemistry and Biological Chemistry, School of Physical and Mathematical Sciences, Nanyang Technological University, 21 Nanyang Link, Singapore 637371, ^2^Institute of Bioorganic Chemistry, Polish Academy of Sciences, Noskowskiego 12/14, 61-704 Poznan, Poland and ^3^Division of Structural Biology and Biochemistry, School of Biological Sciences, Nanyang Technological University, 60 Nanyang Drive, Singapore 637551

## Abstract

Triplex is emerging as an important RNA tertiary structure motif, in which consecutive non-canonical base pairs form between a duplex and a third strand. RNA duplex region is also often functionally important site for protein binding. Thus, triplex-forming oligonucleotides (TFOs) may be developed to regulate various biological functions involving RNA, such as viral ribosomal frameshifting and reverse transcription. How chemical modification in TFOs affects RNA triplex stability, however, is not well understood. Here, we incorporated locked nucleic acid, 2-thio U- and 2′-*O* methyl-modified residues in a series of all pyrimidine RNA TFOs, and we studied the binding to two RNA hairpin structures. The 12-base-triple major-groove pyrimidine–purine–pyrimidine triplex structures form between the duplex regions of RNA/DNA hairpins and the complementary RNA TFOs. Ultraviolet-absorbance-detected thermal melting studies reveal that the locked nucleic acid and 2-thio U modifications in TFOs strongly enhance triplex formation with both parental RNA and DNA duplex regions. In addition, we found that incorporation of 2′-*O* methyl-modified residues in a TFO destabilizes and stabilizes triplex formation with RNA and DNA duplex regions, respectively. The (de)stabilization of RNA triplex formation may be facilitated through modulation of van der Waals contact, base stacking, hydrogen bonding, backbone pre-organization, geometric compatibility and/or dehydration energy. Better understanding of the molecular determinants of RNA triplex structure stability lays the foundation for designing and discovering novel sequence-specific duplex-binding ligands as diagnostic and therapeutic agents targeting RNA.

## INTRODUCTION

**Figure 1.**
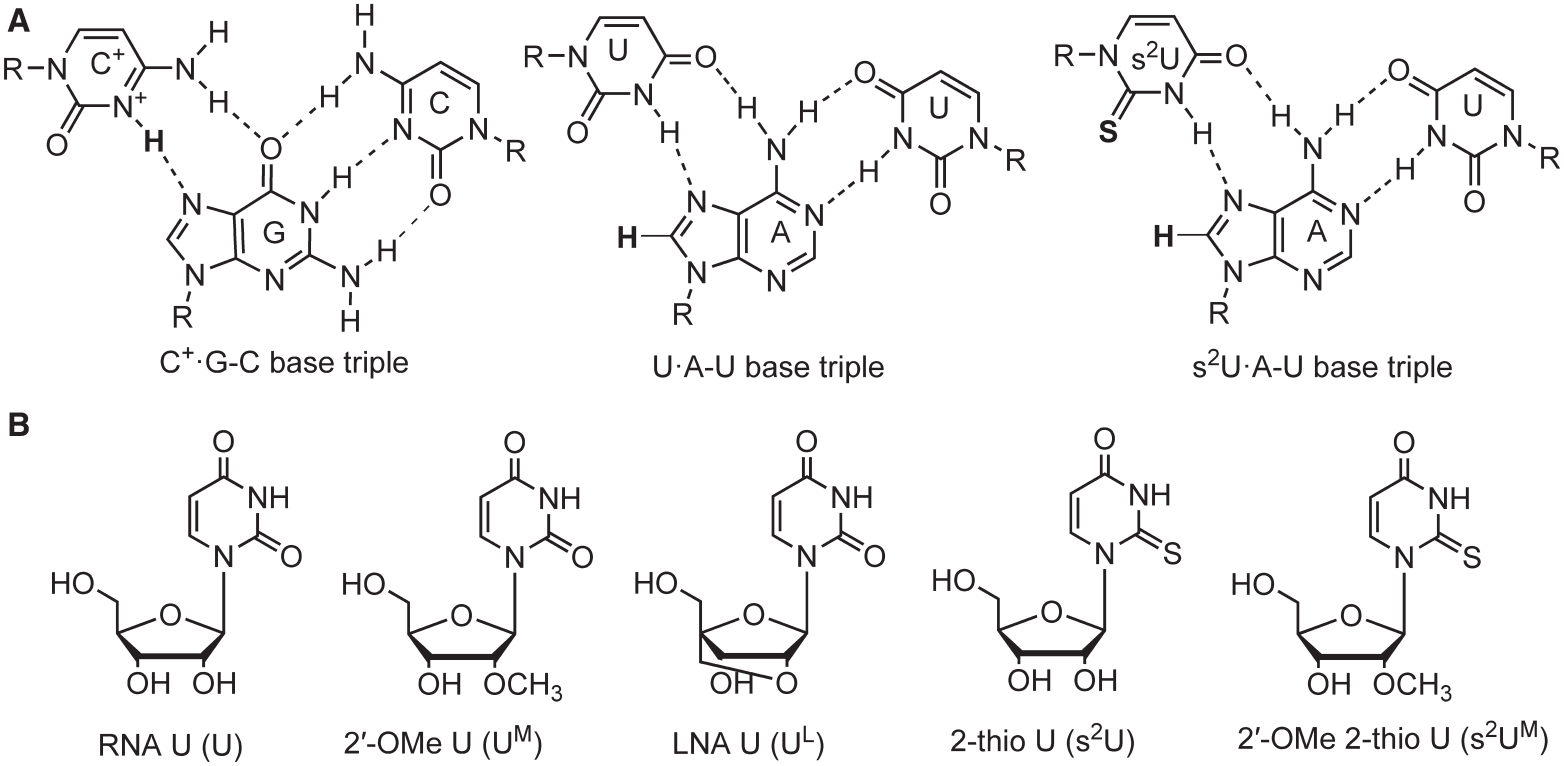
(**A**) Base triples studied in this article. The N3 atom of C base needs to be protonated (shown in bold) to form C^+^·G–C base triple. The s^2^U·A–U base triple is more stable than unmodified U·A–U base triple because of enhanced van der Waals interaction between the thio group in 2-thio U and H8 hydrogen (shown in bold) in A and other molecular interactions. (**B**) Chemical structures of nucleosides: RNA U (U), 2′-OMe U (U^M^), LNA U (U^L^), 2-thio U (s^2^U) and 2′-OMe 2-thio U (s^2^U^M^).

The biological functions of RNAs include gene regulation, catalysis, immunomodulation and acting as templates for the synthesis of protein, RNA and DNA. Recent advances in understanding the structures, energetics and functions of RNAs provide the foundation for developing nucleic acid-based diagnostics, therapeutics and nanobiotechnologies (1–7). Triplex is emerging as an important RNA tertiary structure motif, in which consecutive non-canonical base pairs (see Figure 1A for example) form between a duplex and a third strand (8–18). RNA duplex region is also often functionally important site for protein binding (19–25). Thus, there is a great potential to design and discover chemically modified triplex-forming oligonucleotides (TFOs) and other therapeutic ligands targeting RNA duplex regions, via reprogramming of RNA–RNA and RNA–protein interactions.

**Figure 2.**
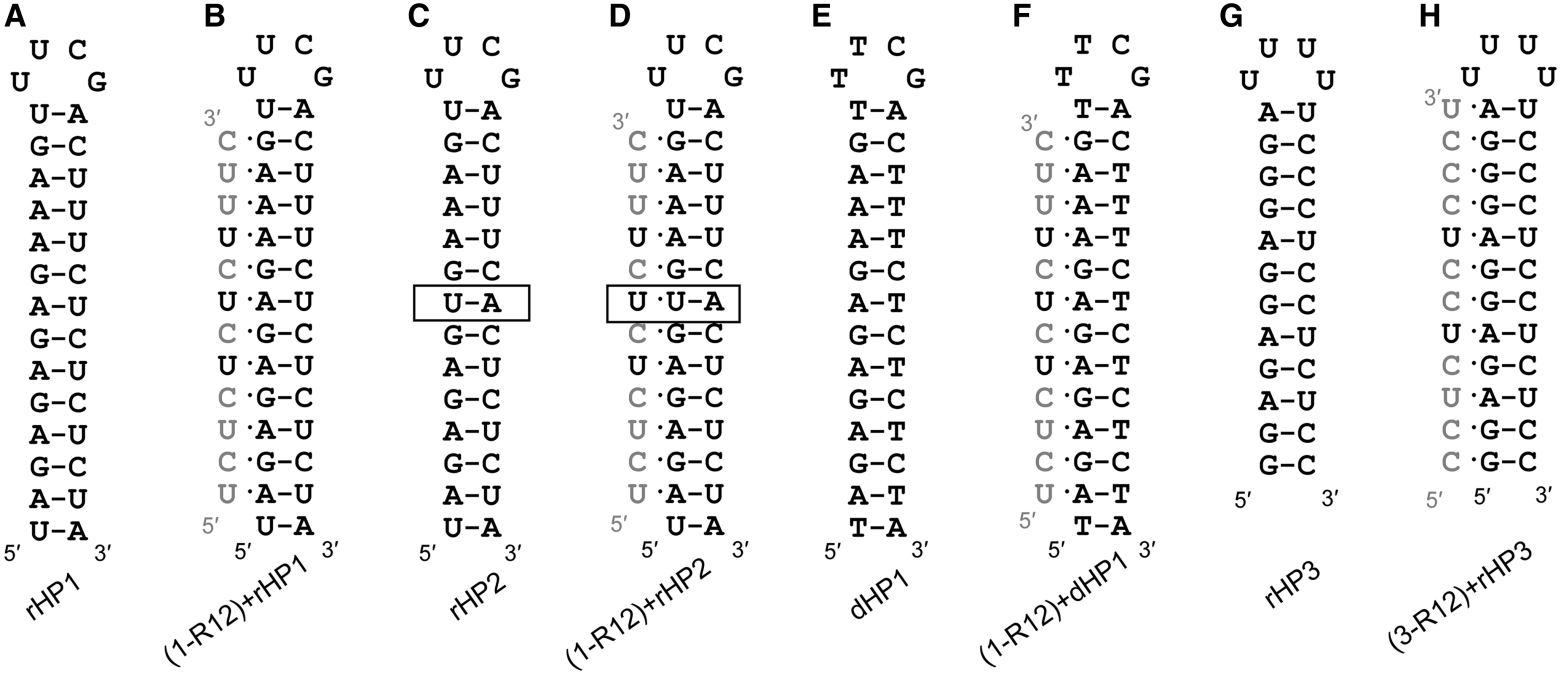
Hairpin and triplex structures studied in this article. Representative TFOs’ strands (1-R12 or 3-R12) are shown in gray with the residues to be modified shown in black. (**A**) A 14-bp 32-nt RNA hairpin (rHP1). (**B**) A triplex formed between TFO 1-R12 and rHP1. (**C**) A mutated (U–A inversion, shown in box) 14-bp 32-nt RNA hairpin (rHP2). (**D**) A hypothetical triplex formed between non-complementary TFO 1-R12 and rHP2. A mismatched base triple (U·U–A) is indicated. (**E**) A 14-bp 32-nt DNA hairpin (dHP1), which is homologous to rHP1. (**F**) A triplex formed between TFO 1-R12 and dHP1. (**G**) A 12-bp 28-nt RNA hairpin (rHP3). (**H**) A triplex formed between TFO 3-R12 and rHP3.

Formation of major-groove pyrimidine–purine–pyrimidine triplexes with parental DNA duplexes (Figures 1 and 2) facilitates sequence-specific DNA cleavage (26,27) and inhibition of transcription (28). Thus, extensive studies have been carried out to use chemically modified TFOs to enhance the binding affinity with DNA duplexes (29,30). For example, 2′-*O* methyl (2′-OMe) (Figure 1B) modification in TFOs favors triplex formation (31,32) with parental DNA duplexes. Incorporation of locked nucleic acid (LNA) [also known as 2′,4′-bridged nucleic acid (2′,4′-BNA)] monomers (Figure 1B) significantly enhances triplex formation with DNA duplexes (33–35). A base modification, 2-thio U (Figure 1B) in TFOs, is effective in stabilization of triplexes with parental DNA duplexes (36).

Surprisingly, limited studies have been reported in targeting RNA duplex regions by triplex formation. To the best of our knowledge, it is not known how incorporation of LNA and 2-thio U-modified residues into TFOs affects RNA triplex stability. Here, we incorporated LNA U (U^L^), 2-thio U (s^2^U), 2′-OMe 2-thio U (s^2^U^M^), 2′-OMe U (U^M^) and 2′-OMe C (C^M^) residues into 12-nt all pyrimidine RNA TFOs, and we studied the major-groove triplex formation of the modified TFOs with RNA and DNA hairpins (Figure 2) by ultraviolet (UV)-absorbance-detected thermal melting at various NaCl concentrations and pH’s. The RNA triplex formation was further tested by gel electrophoresis and surface plasmon resonance (SPR) experiments.

## MATERIALS AND METHODS

### Oligonucleotides

Chemically synthesized and purified DNA hairpin 1 (dHP1), RNA hairpin 1 (rHP1), RNA hairpin 2 (rHP2), RNA hairpin 3 (rHP3), biotin-labeled RNAs and unmodified RNA TFOs (Figure 2) were purchased from Sigma-Aldrich in Singapore. Chemically modified TFOs were chemically synthesized, deprotected and purified as reported (37–39). All oligonucleotides were characterized by electrospray ionization mass spectrometry (ESI-MS). The sequences of TFOs are listed in Table 1.

**Table 1.** TFO sequences studied in this article

TFO	Sequence (5′–3′)^a^											
1-D12^b^	T	C	T	C	T	C	T	C	T	T	T	C
1-R12	U	C	U	C	U	C	U	C	U	U	U	C
Control	A	U	C	U	G	U	U	C	C	A	C	U
1-RU^L^1	U	C	U	C	U	C	U^L^	C	U	U	U	C
1-RU^L^2	U	C	U	C	U^L^	C	U^L^	C	U	U	U	C
1-RU^L^3	U	C	U	C	U^L^	C	U^L^	C	U^L^	U	U	C
1-Rs^2^U1	U	C	U	C	U	C	s^2^U	C	U	U	U	C
1-Rs^2^U2	U	C	U	C	s^2^U	C	s^2^U	C	U	U	U	C
1-Rs^2^U3	U	C	U	C	s^2^U	C	s^2^U	C	s^2^U	U	U	C
1-RU^M^3	U	C	U	C	U^M^	C	U^M^	C	U^M^	U	U	C
1-M12	U^M^	C^M^	U^M^	C^M^	U^M^	C^M^	U^M^	C^M^	U^M^	U^M^	U^M^	C^M^
1-MU^L^1	U^M^	C^M^	U^M^	C^M^	U^M^	C^M^	U^L^	C^M^	U^M^	U^M^	U^M^	C^M^
1-MU^L^2	U^M^	C^M^	U^M^	C^M^	U^L^	C^M^	U^L^	C^M^	U^M^	U^M^	U^M^	C^M^
1-MU^L^3	U^M^	C^M^	U^M^	C^M^	U^L^	C^M^	U^L^	C^M^	U^L^	U^M^	U^M^	C^M^
1-Ms^2^U^M^1	U^M^	C^M^	U^M^	C^M^	U^M^	C^M^	s^2^U^M^	C^M^	U^M^	U^M^	U^M^	C^M^
1-Ms^2^U^M^2	U^M^	C^M^	U^M^	C^M^	s^2^U^M^	C^M^	s^2^U^M^	C^M^	U^M^	U^M^	U^M^	C^M^
1-Ms^2^U^M^3	U^M^	C^M^	U^M^	C^M^	s^2^U^M^	C^M^	s^2^U^M^	C^M^	s^2^U^M^	U^M^	U^M^	C^M^
3-R12	C	C	U	C	U	C	C	U	C	C	C	U
3-Rs^2^U2	C	C	U	C	s^2^U	C	C	s^2^U	C	C	C	U
3-RU^L^2	C	C	U	C	U^L^	C	C	U^L^	C	C	C	U

^a^All listed sequences have RNA residues unless otherwise noted. The modified nucleotides studied are LNA U (U^L^), 2-thio U (s^2^U), 2′-OMe U (U^M^), 2′-OMe C (C^M^) and 2′-OMe 2-thio U (s^2^U^M^). In the nomenclature for TFOs, the first number (1 or 3) indicates the TFO’s complementary hairpin sequence (rHP1, dHP1 or rHP3), and the last number (1, 2, 3 or 12) indicates the number of modified residues. The first letters (D, R and M) indicate the main backbone compositions of DNA, RNA and 2′-OMe RNA, respectively.

^b^In TFO 1-D12, all 12 residues are unmodified deoxyribonucleotides.

### UV-absorbance-detected thermal melting

All UV absorbance versus temperature thermal melting studies were carried out using a Beckman DU 800 spectrophotometer connected to a computer for data collection and analysis. High-performance transport and multiple-cell holder were used. The temperature was increased from 15 to 95°C and then decreased back to 15°C at a ramp rate of 0.2°C/min (or 1°C/min for duplexes formed between TFO strands and a 12-nt all purine strand, 5′-AGAGAGAGAAAG-3′) with a Peltier temperature controller, and the absorbance at 260 or 265 nm (only for 1-Rs^2^U3) was recorded every 0.5°C. The samples for UV-absorbance-detected thermal melting studies contained 5 µM hairpin and 5 µM TFO in 100–1000 mM NaCl, 0.5 mM ethylenediaminetetraacetic acid (EDTA), 20 mM 2-morpholinoethanesulfonic acid (MES) (pH 5.0, 5.5 and 6.0) or 20 mM 4-(2-Hydroxyethyl)piperazine-1-ethanesulfonic acid (HEPES) (pH 6.5, 6.8 and 7.0). We chose MES and HEPES buffers because their p*K*_a_ values are relatively independent of temperature (40). A detailed triplex annealing protocol can be found in the Supplementary Data. The first derivative curves were fit to Gaussian functions, and the temperatures with maximum first derivatives (at Gaussian peaks) in the melting (heating) curves were taken as the melting temperatures (*T*_m1_ for triplex to hairpin transition and *T*_m2_ for hairpin to single-strand transition). Hysteresis between heating and cooling curves was observed for triplex to hairpin transitions. No hysteresis was observed for hairpin to single-strand transition. Equilibrium thermodynamic parameters for the triplex to hairpin transitions were not obtained because of the hysteresis. Equilibrium thermodynamic parameters for the duplex and hairpin formation were obtained by fitting to a two-state model with the MeltWin program (41).

### Gel electrophoresis

The native polyacrylamide gel electrophoresis experiments were performed in 16.5- × 22-cm gel containing 12% acrylamide (acrylamide/Bis–acrylamide = 19:1) at 4°C. RNA hairpin and TFOs were both 1 µM, which were incubated in 40 µl of buffer of 100 mM NaCl, 20 mM MES and 0.5 mM EDTA at pH 5.5, and left for 3 h before loading. Eight microliters of 35% glycerol was then added into 40 µl of loading buffer. The running buffer contains 10 mM NaCl, 2 mM MgCl_2_ and 1× TAE (40 mM Tris–acetate and 1 mM EDTA) at pH 6. The gel electrophoresis experiments were run at 160 V for 12–16 h, and the gels were stained by ethidium bromide, and the hairpin and triplex bands were imaged by a Typhoon scanner (GE Healthcare).

### Surface plasmon resonance assay

All SPR experiments were run with a constant flow (10 μl/min) of running buffer (100 mM NaCl, 0.5 mM EDTA and 20 mM MES, pH 5.5) on a Biacore T200 (BIAcore AB, GE Healthcare) with a carboxymethylated dextran-coated sensor chip (CM5-S) at 25°C. The surfaces were first activated for 7 min with 1:1 mixture of 0.2 M *N*-ethyl-*N*′-[3-(diethylamino)propyl]carbodiimide and 50 mM *N*-hydroxysuccinimide. Neutravidin was then dissolved into 10 mM sodium acetate at pH 6.0 and immobilized at 10 µl/min flow rate onto the surfaces by standard amine coupling procedure to achieve 4000 RU. The surfaces were finally blocked with 0.5 M ethanolamine–HCl at pH 8.5 for 7 min. Biotinylated RNA hairpin (5′-biotin-TTTTGGAGAGGAGGGAUUUUUCCCUCCUCUCC, with four DNA thymine (T) residues incorporated as a linker between biotin and RNA hairpin, rHP3) and an RNA hairpin control (5′-biotin-TTTTUAGAGAGAGAAAGUUUCGACUUUCUCUCUCUA, with four DNA thymine (T) residues incorporated as a linker between biotin and RNA hairpin, rHP1) were captured on sensor chip surfaces to ∼740 RU. Serially diluted TFOs (3-R12, 3-Rs^2^U2, and 3-RU^L^2 at 82 nM, 247 nM, 741 nM, 2.2 µM, 6.7 µM and 20 µM) were injected (10 µl/min) for 10 min across the surfaces with immobilized RNA hairpins. After a dissociation period (1200 s), a 60 s pulse of 0.1% sodium dodecyl sulfate in H_2_O was applied to regenerate the surfaces, followed by a 10-min running buffer flow. All the sensorgrams were corrected by subtraction of the buffer blanks and responses of TFOs on the RNA hairpin control surface. Processed data were globally analyzed and fit to a simple 1:1 interaction model with mass transport coefficient.

## RESULTS AND DISCUSSION

### Effect of pH on RNA triplex formation

**Figure 3.**
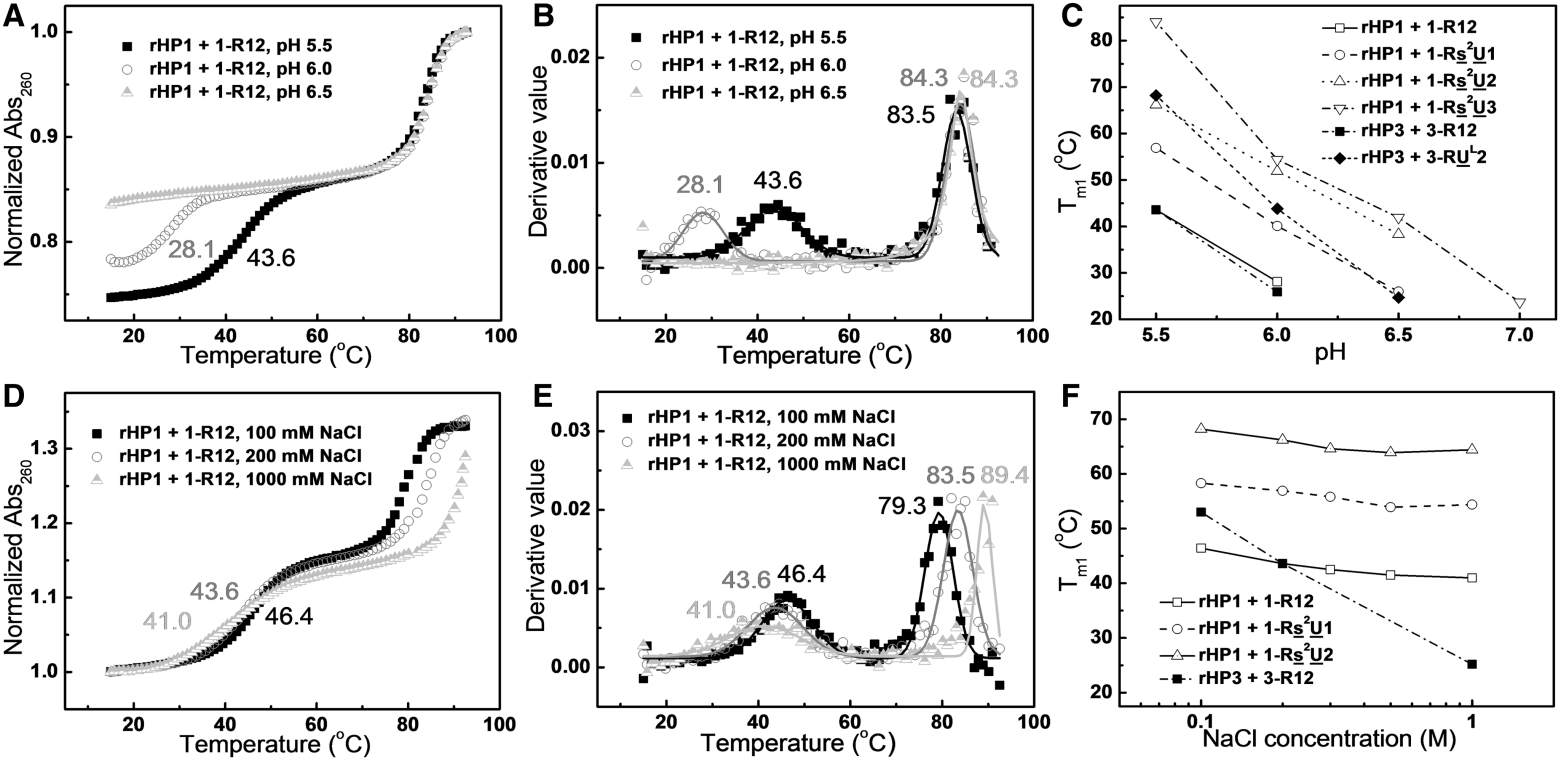
Effects of pH and NaCl concentration on RNA triplex thermal stability. (**A**) UV-absorbance-detected thermal melting curves of TFO 1-R12 binding to the RNA hairpin (rHP1, Figure 2A) at pH 5.5, 6.0 and 6.5 in 200 mM NaCl. (**B**) First derivatives of thermal melting curves in (A) reveal *T*_m1_ values of 43.6, 28.1 and <20°C, respectively, at pH 5.5, 6.0 and 6.5. (**C**) Melting temperatures of triplexes (*T*_m1_) at various pH’s in 200 mM NaCl. (**D**) Thermal melting curves of TFO 1-R12 binding to rHP1 at pH 5.5 in varying NaCl concentrations. (**E**) First derivatives of thermal melting curves in (D). With increasing NaCl concentration, triplex melting temperature (*T*_m1_) decreases, whereas hairpin (rHP1) melting temperature (*T*_m2_) increases. (**F**) Melting temperatures of triplexes (*T*_m1_) at pH 5.5 in different NaCl concentrations.

Cytosine N3 atoms in a TFO need to be protonated to form hydrogen bonds (Hoogsteen base pairs) with guanine N7 atoms in the purine strand of a duplex (Figures 1 and 2). Thus, we investigated the effect of pH on triplex formation. Our thermal melting studies for RNA triplexes formed between TFOs and RNA hairpins rHP1 and rHP3 (Figure 2A and G) reveal that the melting temperatures of the RNA hairpins (*T*_m2_) do not change significantly with pH (Figure 3A and B and Supplementary Table S1), consistent with previous studies for RNA duplex structures (42). The melting temperatures of the RNA triplexes (*T*_m1_) decrease significantly (by >20°C) with increasing pH (from pH 5.5 to 7.0) (Figure 3A–C and Supplementary Table S1), which is consistent with a p*K*_a_ of ∼7.0 for cytosine N3 atoms in TFOs of major-groove RNA triplexes (9). Because of the hysteresis observed in triplex to hairpin transitions (see Supplementary Figure S3 for example), we did not obtain equilibrium thermodynamic parameters.

### Effect of salt concentration on RNA triplex formation

With increasing concentration of NaCl from 100 mM to 1 M, the melting temperature of the RNA hairpin (*T*_m2_) increases as expected, whereas the melting temperatures of the RNA triplexes (*T*_m1_) decrease modestly (Figure 3D–F and Supplementary Table S1). Destabilization of triplexes with increasing concentration of NaCl is probably because of the fact that increasing concentration of NaCl decreases the p*K*_a_ of N3 of cytosines in the TFOs, which in turn destabilizes the RNA triplexes at pH 5.0–7.0. Our results are consistent with the previous finding that Na^+^ and H^+^ are competitive in binding to unmodified DNA triplexes containing both C^+^·G–C and T·A–T base triples (43–45). Because of the fact that formation of C^+^·G–C base triples results in the release of Na^+^, a stronger salt-dependent triplex thermal stability is observed for rHP3 (eight G–C pairs, Figure 2B) than for rHP1 (five G–C pairs, Figure 2H) (Figure 3F). The pH and salt dependence measurements facilitate the interpretation of the chemical modification results (see later in the text).

### Incorporation of LNA U and 2-thio U in a TFO enhances its binding to an RNA duplex region

**Figure 4.**
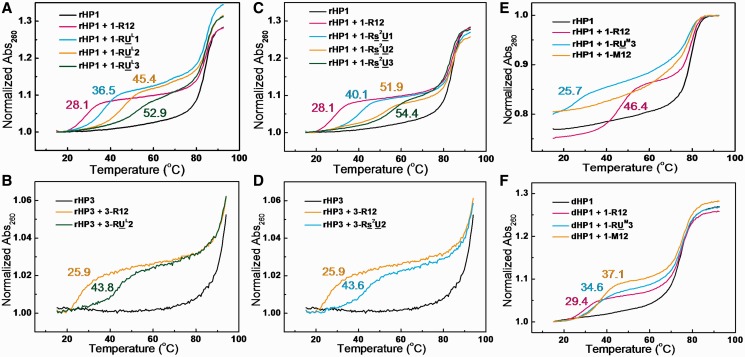
Effects of TFO base and sugar modifications on RNA triplex thermal stability in 200 mM NaCl at pH 6.0 unless otherwise noted. (**A** and **B**) UV-absorbance-detected thermal melting curves of LNA-modified TFOs binding to rHP1 and rHP3. First derivatives of thermal melting curves reveal *T*_m1_ values of 28.1, 36.5, 45.4 and 52.9°C, respectively, for 1-R12, 1-RU^L^1, 1-RU^L^2 and 1-RU^L^3. *T*_m1_ values are 25.9 and 43.8°C, respectively, for 3-R12 and 3-RU^L^2 binding to rHP3. (**C** and **D**) The 2-thio U-modified TFOs binding to rHP1. The absorption wavelength for 1-Rs^2^U3 binding to rHP1 was taken at 265 nm. *T*_m1_ values are 28.1, 40.1, 51.9 and 54.4°C, respectively, for 1-R12, 1-Rs^2^U1, 1-Rs^2^U2 and 1-Rs^2^U3. *T*_m1_ values are 25.9 and 43.6°C, respectively, for 3-R12 and 3-Rs^2^U2 binding to rHP3. (**E**) Incorporation of 2′-OMe residues in TFOs destabilizes (RNA)_2_–(TFO) triplex formation. The 2′-OMe-modified TFOs binding to rHP1 in 100 mM NaCl at pH 5.5. *T*_m1_ values are 46.4, 25.7 and <20°C, respectively, for 1-R12, 1-RU^M^3 and 1-M12. (**F**) Incorporation of 2′-OMe residues in TFOs stabilize (DNA)_2_–(TFO) triplex formation. The 2′-OMe-modified TFOs binding to dHP1 in 200 mM NaCl at pH 6.5. *T*_m1_ values are 29.4, 34.6 and 37.1°C, respectively, for 1-R12, 1-RU^M^3 and 1-M12.

LNA modification (Figure 1B) pre-organizes oligonucleotides in an A-form-like structure by locking the sugar pucker in C3′-endo conformation and thus favors the formation of duplexes with complementary single-strand DNA or RNA and the formation of triplexes with parental DNA duplexes (33–35,38,46,47). It is known that C3′-endo sugar pucker in TFOs is favored in both RNA and DNA triplex formation (14,15,48–50). But how LNA incorporation in TFOs affects binding to an RNA duplex region is not known. Our thermal melting results (Figure 4A and Supplementary Table S1) reveal that LNA U-modified RNA TFOs enhance triplex thermal stability with parental RNA duplex structure segment within a hairpin (rHP1, Figure 2A). Triplexes with more uridines modified are more stable than those with less uridines modified (Figure 4A and Supplementary Table S1). The *T*_m1_ for the triplex with unmodified RNA TFO (1-R12) is 28.1°C in 200 mM NaCl at pH 6.0. At the same buffer condition, the *T*_m1_’s for the triplexes with modified TFOs with one, two and three LNA U residues (1-RU^L^1, 1-RU^L^2 and 1-RU^L^3) are 36.5, 45.4 and 52.9°C, respectively. Similar stabilizing effect is observed for the complementary TFO (3-RU^L^2 versus 3-R12) binding to rHP3 (Figure 4B). rHP3 is only partially melted at ∼90°C, consistent with previously reported results (51,52).

**Figure 5.**
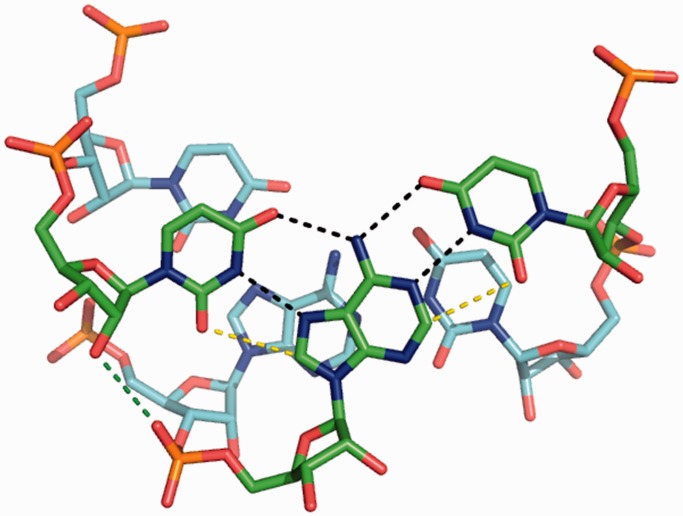
Stacking of two RNA U·A–U base triples (taken from PDB 3P22). Hydrogen bonds and van der Waals contacts (dashed lines) are shown only for the top base triple. The U·A–U base triple is stabilized by base–base hydrogen bonds (black dashed lines) and van der Waals contacts (yellow dashed lines and stacking interaction), all of which are enhanced by 2-thio U modification in the TFO strand. Base triples are also stabilized by hydrogen bonds between 2′-hydroxyl groups in the third strand and non-bridging oxygen in the purine strands of the RNA duplex (green dashed line). The 2′-OMe U modification in the third strand (TFO strand) disrupts this hydrogen bond, and the methyl group may cause steric clash with the backbone of the purine strand. LNA U modification in the TFO strand may not cause steric clash because of the fact that the methylene group is highly constrained.

Each LNA modification increases *T*_m1_ by ∼9°C (Figure 4A and B). Thus, stabilization effect of LNA incorporation is relatively position and sequence environment independent. We note that LNA modification disrupts a potential hydrogen bond between 2′-OH in the TFO and a non-bridging oxygen in the purine strand of a duplex (Figure 5) (14,15,48,53,54). The importance of this hydrogen bond is corroborated by the fact that a DNA TFO (1-D12, Table 1) does not bind to rHP1 (Supplementary Figure S1A), consistent with previous results (51,55). Thus, the energy penalty because of the loss of the hydrogen bonds is compensated by the pre-organization of TFO backbone. Thus, our results suggest that LNA may be incorporated into TFOs to enhance binding to RNA duplex regions.

The 2-thio U modification (Figure 1) in an oligonucleotide stabilizes the formation of RNA duplexes with complementary single-strand DNA or RNA and the formation of triplexes with a parental DNA duplex, respectively (36–38,49). Our thermal melting results reveal that incorporation of 2-thio U in a TFO also significantly stabilizes a triplex with a parental RNA duplex structure segment within a hairpin (rHP1, Figure 2A). The *T*_m1_’s for the triplexes with one, two and three 2-thio U residues (1-Rs^2^U1, 1-Rs^2^U2 and 1-Rs^2^U3) in the TFOs are 40.1, 51.9 and 54.4°C, respectively, in 200 mM NaCl at pH 6.0 (Figure 4C and Supplementary Table S1). Similar stabilizing effect is also observed for the complementary TFO (3-Rs^2^U2 versus 3-R12) binding to rHP3 (Figure 4D).

The stabilization effect of 2-thio U modification in a TFO is probably because of the fact that the steric repulsion between the 2′-hydroxyl group of the ribose and the bulky 2-thio group of 2-thio U favors C3′-endo conformation of the ribose (56), which facilitates stable triplex formation (48). Base–base hydrogen bonding interaction (between the imino proton H3 in 2-thio U and N7 in A) is also enhanced with decreased p*K*_a_ of N3 (from 9.3 to 8.8) on thiolation of U (49,57). In addition, 2-thio U modification enhances TFO binding by reduced thermodynamic cost of dehydration and improved van der Waals contact between sulfur atom in 2-thio U and H8 hydrogen in A (Figures 1A and 5) (37,58).

Moreover, the enhanced triplex stability may be explained in terms of the strong stacking effect of the 2-thio group with adjacent bases (Figure 5) (36). Base–base stacking interactions are sequence environment dependent (42,43,59,60), which may explain one extra 2-thio U modification in 1-Rs^2^U3, which is flanked by C and U (Table 1), increases *T*_m1_ by only ∼3°C relative to 1-Rs^2^U2 (Figure 4C). In contrast, the 2-thio U modifications present in 1-Rs^2^U1, 1-Rs^2^U2 and 3-Rs^2^U2 are all flanked by two C’s, with each modification increasing *T*_m1_ by ∼10°C (Figure 4C and D). The 2-thio U is a naturally occurring modification in RNA (61). Thus, nature may have evolved to use the conservative atomic mutation (2-thio U) to modulate RNA secondary and tertiary structure stability and function.

**Figure 6.**
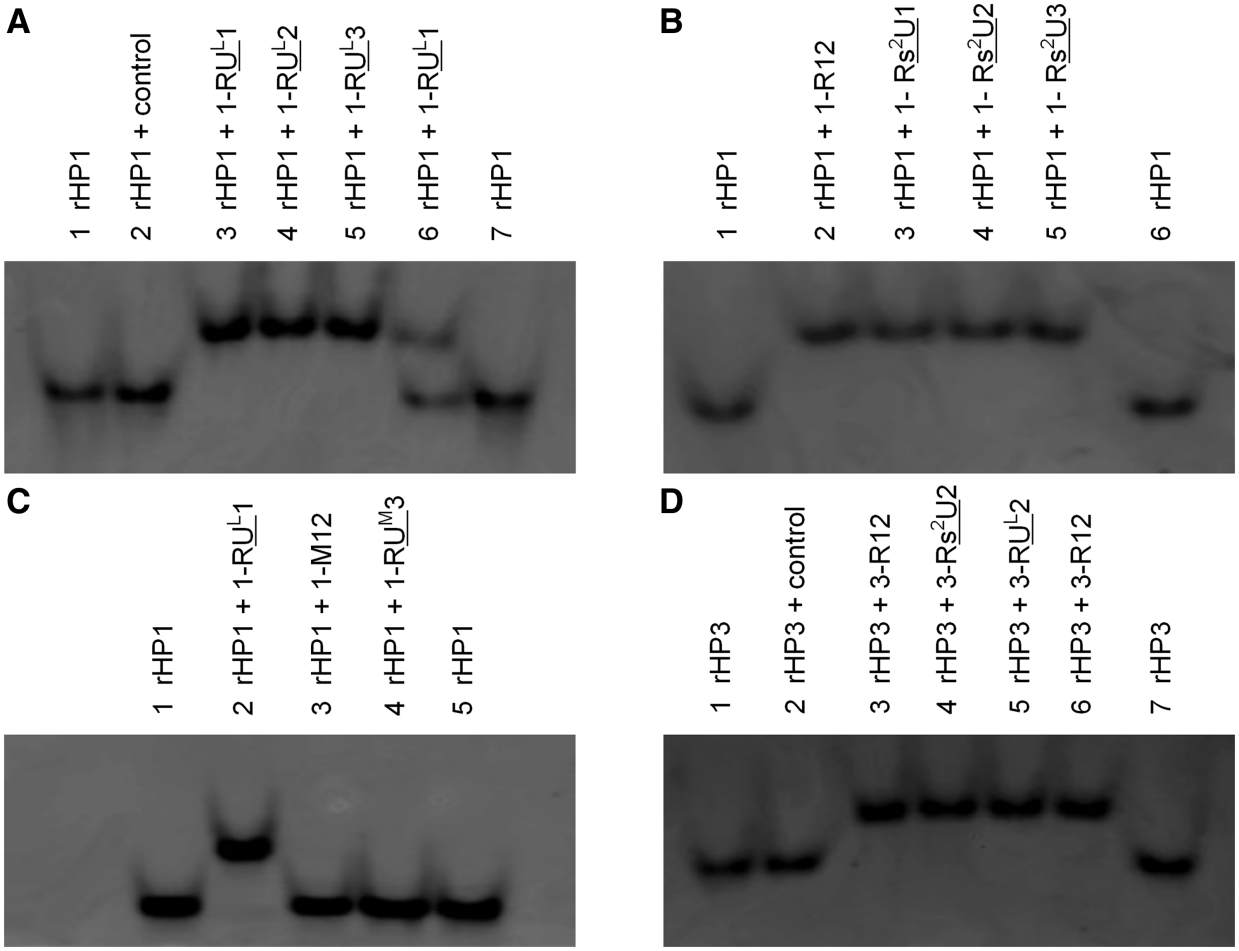
Native polyacrylamide gel for the RNA hairpins (rHP1 and rHP3) and triplexes. (**A**) rHP1 binds to modified RNA TFOs incorporated with LNA U residues. In lane 2, the control TFO (Table 1) does not bind to rHP1. In lane 6, rHP1 to 1-RU^L^1 molar ratio is 1:0.5. The fact that only two bands (rHP1 and triplexes) were observed suggests that the designed RNA triplexes form without alternative structures. (**B**) rHP1 binds to unmodified and modified RNA TFOs incorporated with 2-thio U residues. (**C**) The 2′-OMe modifications in TFOs destabilize RNA triplex formation. RNA triplex bands were not observed for TFOs incorporated with three or more 2′-OMe residues. (**D**) rHP3 binds to unmodified and modified TFOs incorporated with 2-thio U and LNA U residues.

We confirmed triplex formation by native polyacrylamide gel electrophoresis experiment (Figure 6). The gels were post-stained by ethidium bromide, which intercalates into hairpin and triplex structures. As expected, the RNA hairpin bands moved faster than triplexes. In the lane 2 shown in Figure 6A and D, rHP1 and rHP3 were mixed with an RNA TFO control sequence (5′-AUCUGUUCCACU-3′, Table 1), which is not sequence complementary to rHP1or rHP3 to form triplexes. As expected, RNA triplex did not form between the RNA hairpins and RNA TFO control. Two bands were observed in lane 6 (Figure 6A) with a molar ratio of rHP1 to TFO (1-RU^L^1) at 1:0.5. The result suggests that a mixture of ∼50% triplex and ∼50% RNA hairpin is present in lane 6 (Figure 6A). Direct imaging of the all pyrimidine TFO bands by staining with Syber Green II was tested, but we observed only hairpin and triplex bands. Consistently, no apparent thermal melting transitions were observed for TFOs alone (data not shown). We did observe a band for an all purine 12-nt single-strand (5′-AGAGAGAGAAAG-3′, Supplementary Figure S2C and D) by both ethidium bromide and Syber Green II staining (data not shown). Taken together, our thermal melting and gel results suggest that the designed RNA triplex structures (Figure 2B and H) form for unmodified RNA TFO and modified RNA TFOs incorporated with LNA U and 2-thio U residues. In addition, we carried out SPR experiment to monitor the triplex formation in real-time (Supplementary Figure S4). The SPR results suggest that the designed triplex structures (Figure 2H) indeed form.

### Binding specificity assay

We further tested the binding of the RNA TFOs to an RNA hairpin with one A–U base pair inverted compared with rHP1 (rHP2, Figure 2C and D). Our thermal melting results in 200 mM NaCl at varying pH reveal that the triplex melting temperatures (*T*_m1_) are <20°C at pH 6.0 (Supplementary Table S1). We tested TFOs 1-RU^L^1 and 1-Rs^2^U3 binding to rHP2 at low pH (5.5 and 5.0), and *T*_m1_ values were found to be >30°C lower than those of sequence-complementary triplexes when *T*_m1_ and *T*_m2_ are not overlapped (Supplementary Tables S1). The native gel results further confirm that triplexes do not form between rHP2 and TFOs with one mismatch (Supplementary Figure S1F–H). The results indicate that the TFOs have good sequence specificity in binding to an RNA duplex region.

### Incorporation of 2′-OMe residues in a TFO destabilizes its binding to an RNA duplex region

We then investigated how 2′-OMe modification (Figure 1B) in a TFO affects the thermal stability of a triplex with a parental RNA duplex region (Figure 2B). The *T*_m1_ of triplex with TFO 1-RU^M^3 (25.7°C), which has three 2′-OMe residues, is lower than that with RNA TFO (1-R12) (46.4°C) in 100 mM NaCl at pH 5.5 (Figure 4E and Supplementary Table S1). All the complementary modified TFOs with more than three 2′-OMe modified residues do not bind to rHP1 (Figure 4E and Supplementary Table S1). No obvious gel electrophoresis mobility difference was seen between rHP1 alone and rHP1 mixed with TFOs with three or more 2′-OMe residues (Figure 6C and Supplementary Figure S1A and B), further suggesting that 2′-OMe TFOs do not bind to rHP1. The results indicate that 2′-OMe-modified TFOs destabilize triplex formation with RNA duplex region.

It is probable that substitution of 2′-OH with 2′-OMe disrupts the hydrogen bond between 2′-OH in the TFO and a non-bridging oxygen in the purine strand of a duplex (Figure 5) (14,15,48,53,54) and thus disfavors RNA triplex formation (32). In addition, unlike the highly restrained methylene group in LNA, the exposed methyl group in 2′-OMe RNA (Figure 1B) in the TFO may cause steric clash with the narrow major groove of the RNA duplex region.

### TFOs binding to a homologous DNA duplex region

Thermodynamically stable major-groove triplex structure formation without chemical modification is limited to a pyrimidine TFO strand binding to a duplex region with purines on one strand and pyrimidines on the other (50,62–64). Previous thermodynamic studies reveal that to form stable triplexes, RNA is preferred on both pyrimidine strands, whereas DNA is preferred on the purine strand (51). If the purine strand of a parental duplex region is RNA, only RNA TFO strand binds tightly to the parental duplex region (55).

To study the effect of target strand composition on triplex formation, we measured the binding of the complementary TFOs to a DNA hairpin target (dHP1, Figure 2E and F) with the same sequence as the RNA hairpin (rHP1, Figure 2A and B). Our thermal melting results reveal that the triplex melting temperatures (*T*_m1_) are always higher with a DNA duplex region than with a homologous RNA duplex region (ΔT _m1_ ranging from 6 to 21°C in 200 mM NaCl at pH 6.0) (Supplementary Table S1). The DNA hairpin, dHP1, has a melting temperature (*T*_m2_) of ∼74°C in 200 mM NaCl, which is ∼10°C lower than the RNA hairpin, rHP1 (Figure 4, Supplementary Table S1), consistent with the prediction from nearest-neighbor models (59,65,66).

The relatively wider major groove of a DNA duplex (compared with an RNA duplex) may provide relatively easier access for TFO binding. Consistently, incorporation of 2′-OMe residues in TFOs was found to enhance binding to the DNA hairpin, dHP1 (Figure 4F and Supplementary Table S1), which is in contrast to the significant destabilization effect observed in TFOs binding to the RNA hairpin, rHP1 (see earlier in the text, Figure 4E and Supplementary Table S1). It is likely that the major groove of a DNA duplex is more geometrically compatible for accommodating a 2′-OMe-modified TFO. The results suggest that rules for enhancing DNA triplex formation may not be applicable for RNA triplex formation. One may simply incorporate 2′-OMe or other relatively bulky residues in a TFO to selectively target a DNA duplex over a homologous RNA duplex region.

### The hairpin rHP1 forms without alternative structures

As evidenced by the strand concentration independent *T*_m2_ of rHP1 (Supplementary Table S3), the designed hairpin structure forms without appreciable bimolecular duplex (with an internal loop). It is likely that chemically modified TFOs may displace the pyrimidine segment of the parental RNA hairpin to form parallel or antiparallel duplex structure as shown in Supplementary Figure S2A and B. To test the possibility of the formation of such alternative structures because of strand invasion, we carried out thermal melting studies for the duplexes formed between a 12-nt all purine strand (5′-AGAGAGAGAAAG-3′, Supplementary Figure S2C and D) and various TFOs. The results reveal that most of the duplex formation is relatively pH independent (Δ*T*_m_ is within 3°C from pH 7.0 to 5.5) and have lower *T*_m_ than that of the parental RNA hairpin, rHP1 (Supplementary Figure S5 and Supplementary Tables S1 and S2). The pH independence of melting temperatures suggests that 12-bp parallel RNA duplex structures (formed between TFO sequences and an all purine strand, Supplementary Figure S2D) do not form because the stability of parallel DNA duplex is pH dependent (67). Thus, 9-bp antiparallel duplex structures form between the 12-nt all purine strand and TFOs (see Supplementary Figure S2C for example).

The fact that most of the *T*_m_ values of the 9-bp antiparallel duplexes (Supplementary Figure S2C) are in between *T*_m1_ and *T*_m2_ of triplexes (Supplementary Figure S5 and Supplementary Tables S1 and S2) suggests that neither parallel nor antiparallel duplex structure forms due to strand invasion of rHP1 (Supplementary Figure S2A and B). In addition, there are only two bands observed in native gels (Figure 6 and Supplementary Figure S1), further suggesting no appreciable alternative structures form (63). Thus, our designed 12-base-triple triplex structures (Figure 2B) form between rHP1 and complementary unmodified and modified RNA TFOs incorporated with LNA U and 2-thio U residues.

## CONCLUSION

In this study, we have found that both base and sugar modifications in TFOs may modulate the triplex formation targeting RNA duplex regions. We note that both sugar methylation and base thiolation are naturally occurring RNA modifications. The effects of sugar modifications are probably sequence environment independent. The 2′-OMe sugar modification may be selected (by nature) to destabilize RNA base triple formation because of the loss of a hydrogen bond and steric clash. LNA sugar modification in TFOs stabilizes RNA triplex structures because of backbone pre-organization, despite the loss of a hydrogen bond. The 2-thio U base modification in TFOs stabilizes RNA triplex formation in a sequence environment-dependent manner, probably because of the combined effects of enhanced van der Waals contact, hydrogen bonding, base stacking, backbone pre-organization and reduced energy penalty of dehydration. A thio group may be selected (by nature) to enhance van der Waals interaction with the hydrogen atoms in a duplex major groove (H8 in adenine and guanine, H5 and H6 in uracil and cytosine) and other interactions, and thus favor RNA base triple and other tertiary structures. Our results provide useful insights into rational design of more potent and selective triplex-forming ligands targeting biologically important RNA duplex regions. Studies of other chemical modifications in bases and/or sugar–phosphate backbone and detailed sequence-dependent thermodynamic and kinetic characterizations are in progress.

## SUPPLEMENTARY DATA

Supplementary Data are available at NAR Online: Supplementary Tables 1–3, Supplementary Figures 1–6, Supplementary Method and Supplementary References [41,59].

## FUNDING

Start-up grant of Nanyang Technological University (NTU) (to G.C.); NTU internal grant (Singapore Ministry of Education Academic Research Fund Tier 1) (to G.C.); National Institutes of Health [1R03TW008739-01 to E.K. and D.H. Turner]; National Science Center [N N301 788 440 to E.K. and UMO-2011/03/B/NZ1/00576 and UMO-2011/03/B/ST5/01098 to R.K.]. Funding for open access charge: Start-up grant of Nanyang Technological University (NTU) (to G.C.).

*Conflict of interest statement*. None declared.

## Supplementary Material

Supplementary Data
