# Supplementary material for: Recognition of RNA duplexes by chemically modified triplex-forming oligonucleotides
Source: Nucleic Acids Res. 2013 May 8;41(13):6664–73. doi: 10.1093/nar/gkt352 (PMC3711454; doi:10.1093/nar/gkt352)
Supplement: Supplementary Data [file supp_41_13_6664__index.html]

Recognition of RNA duplexes by chemically modified triplex-forming oligonucleotides — Recognition of RNA duplexes by chemically modified triplex-forming oligonucleotides — Supplementary Data 

# Recognition of RNA duplexes by chemically modified triplex-forming oligonucleotides

## Supplementary Data

files

**Files in this Data Supplement:**

- Supplementary Data - docx file
