# Supplementary material for: Recognition of RNA duplexes by chemically modified triplex-forming oligonucleotides
Source: Nucleic Acids Res. 2013 May 8;41(13):6664–73. doi: 10.1093/nar/gkt352 (PMC3711454; doi:10.1093/nar/gkt352)
Supplement: Supplementary Data [file supp_gkt352_nar-02738-f-2012-File008.docx]

**Supplementary Data**

**Recognition of RNA duplexes by chemically modified triplex-forming oligonucleotides**

Yuan Zhou^1^, Elzbieta Kierzek^2^, Zi Ping Loo^1^, Meraldo Antonio^1^, Yin Hoe Yau^3^, York Wieo Chuah^3^, Susana Geifman-Shochat^3^, Ryszard Kierzek^2,*^ and Gang Chen^1,*^

**Triplexes were annealed as follows:**

1. The 1.5 mL microtube containing RNA hairpin was placed inside a heating block for 5 minutes which has been preset to 95 °C.
2. The tube was then transferred inside an ice block for 5 minutes in order to cool the tube to around 0 °C.
3. The hairpin solution was then transferred to a tube with dried triplex-forming oligonucleotide and the final concentration of the triplex is 5 µM.
4. The 1.5 mL microtube with 5 µM RNA triplex was placed inside a heating block for 5 minutes which has been preset to 65 °C.
5. After heating, heat block was powered off and the tube was left in the heat block for about 2 hours, and then the tube was left inside 4 °C refrigerator for at least three hours in order to form stable RNA triplex.
6. RNA triplex was transferred into the Hellma UV quartz cuvette, and then silicon oil was added on the top of the RNA solution. The cuvettes were centrifuged at 4000 rpm for 10 minutes to evenly spread the silicon oil. The oil was added to prevent water evaporation.

**Table S1.** Melting temperatures of triplexes (*T*_m1_) and hairpins (*T*_m2_)*^a^*

| TFO sequence | Duplex target | pH | [NaCl] (mM) | *T*_m1_(°C) | *T*_m2_(°C) |
| --- | --- | --- | --- | --- | --- |
| No TFO | rHP1 | 6.5 | 200 | N/A | 84.1 |
|  | rHP1 | 6.0 | 200 | N/A | 84.0 |
|  | rHP1 | 5.5 | 1000 | N/A | 91.3 |
|  | rHP1 | 5.5 | 500 | N/A | 87.5 |
|  | rHP1 | 5.5 | 300 | N/A | 86.0 |
|  | rHP1 | 5.5 | 200 | N/A | 83.5 |
|  | rHP1 | 5.5 | 100 | N/A | 79.3 |
|  | dHP1 | 6.5 | 200 | N/A | 73.7 |
|  | dHP1 | 6.0 | 200 | N/A | 73.5 |
|  | dHP1 | 5.5 | 200 | N/A | 74.4 |
|  | dHP1 | 5.5 | 100 | N/A | 66.3 |
|  | rHP2 | 6.0 | 200 | N/A | 83.8 |
|  | rHP2 | 5.5 | 200 | N/A | 83.8 |
|  | rHP3 | 7.0 | 200 | N/A | 91.9 |
|  | rHP3 | 6.0 | 200 | N/A | 92.9 |
|  | rHP3 | 5.5 | 100 | N/A | 92.2 |
| 1-R12 | rHP1 | 6.5 | 200 | <20.0 | 84.3 |
|  | rHP1 | 6.0 | 200 | 28.1 | 84.3 |
|  | rHP1 | 5.5 | 1000 | 41.0 | 89.4 |
|  | rHP1 | 5.5 | 500 | 41.5 | 88.0 |
|  | rHP1 | 5.5 | 300 | 42.5 | 86.2 |
|  | rHP1 | 5.5 | 200 | 43.6 | 83.5 |
|  | rHP1 | 5.5 | 100 | 46.4 | 79.3 |
|  | dHP1 | 6.5 | 200 | 29.4 | 74.8 |
|  | dHP1 | 6.0 | 200 | 45.3 | 73.4 |
|  | dHP1 | 5.5 | 100 | (67.5) | (67.5) |
|  | rHP2 | 6.0 | 200 | <20.0 | 84.7 |
| 1-RU^L^1 | rHP1 | 6.5 | 200 | <20.0 | 84.8 |
|  | rHP1 | 6.0 | 200 | 36.5 | 84.6 |
|  | rHP1 | 5.5 | 200 | 57.5 | 82.5 |
|  | rHP1 | 5.0 | 200 | (83.5) | (83.5) |
|  | dHP1 | 6.0 | 200 | 52.3 | 73.6 |
|  | dHP1 | 5.5 | 200 | (73.5) | (73.5) |
|  | dHP1 | 5.0 | 200 | (73.5) | (73.5) |
|  | rHP2 | 6.0 | 200 | <20.0 | 85.4 |
|  | rHP2 | 5.5 | 200 | 26.7 | 83.8 |
|  | rHP2 | 5.0 | 200 | 46.4 | 83.1 |
| 1-RU^L^2 | rHP1 | 7.0 | 200 | <20.0 | 84.6 |
|  | rHP1 | 6.5 | 200 | 31.8 | 85.1 |
|  | rHP1 | 6.0 | 200 | 45.4 | 84.6 |
|  | dHP1 | 6.0 | 200 | 57.0 | 73.6 |
|  | rHP2 | 6.0 | 200 | <20.0 | 84.7 |
| 1-RU^L^3 | rHP1 | 7.0 | 200 | <20.0 | 84.5 |
|  | rHP1 | 6.5 | 200 | 36.6 | 85.3 |
|  | rHP1 | 6.0 | 200 | 52.9 | 84.3 |
|  | dHP1 | 6.0 | 200 | (75.4) | (75.4) |
|  | rHP2 | 6.0 | 200 | <20.0 | 84.5 |
| 1-Rs^2^U1 | rHP1 | 6.5 | 200 | 26.0 | 84.6 |
|  | rHP1 | 6.0 | 200 | 40.1 | 84.5 |
|  | rHP1 | 5.5 | 1000 | 54.4 | 90.9 |
|  | rHP1 | 5.5 | 500 | 53.9 | 87.0 |
|  | rHP1 | 5.5 | 300 | 55.8 | 86.7 |
|  | rHP1 | 5.5 | 200 | 56.9 | 83.5 |
|  | rHP1 | 5.5 | 100 | 58.3 | 80.0 |
|  | dHP1 | 6.0 | 200 | 56.2 | 74.0 |
|  | rHP2 | 6.0 | 200 | <20.0 | 84.2 |
| 1-Rs^2^U2 | rHP1 | 7.0 | 200 | <20.0 | 84.6 |
|  | rHP1 | 6.5 | 200 | 38.3 | 84.0 |
|  | rHP1 | 6.0 | 200 | 51.9 | 84.0 |
|  | rHP1 | 5.5 | 1000 | 64.4 | 90.9 |
|  | rHP1 | 5.5 | 500 | 63.9 | 87.0 |
|  | rHP1 | 5.5 | 300 | 64.6 | 85.9 |
|  | rHP1 | 5.5 | 200 | 66.2 | 83.5 |
|  | rHP1 | 5.5 | 100 | 68.2 | 79.6 |
|  | dHP1 | 6.0 | 200 | 60.0 | 75.0 |
|  | rHP2 | 6.0 | 200 | <20.0 | 84.7 |
| 1-Rs^2^U3 | rHP1 | 7.0 | 200 | 23.7 | 84.9 |
|  | rHP1 | 6.5 | 200 | 41.9 | 84.1 |
|  | rHP1 | 6.0 | 200 | 54.4 | 83.1 |
|  | rHP1 | 5.5 | 1000 | (90.9) | (90.9) |
|  | rHP1 | 5.5 | 500 | (87.0) | (87.0) |
|  | rHP1 | 5.5 | 300 | (86.0) | (86.0) |
|  | rHP1 | 5.5 | 200 | (84.0) | (84.0) |
|  | rHP1 | 5.5 | 100 | (80.2) | (80.2) |
|  | dHP1 | 6.0 | 200 | (74.1) | (74.1) |
|  | rHP2 | 6.0 | 200 | <20.0 | 83.9 |
|  | rHP2 | 5.5 | 200 | 44.9 | 83.6 |
|  | rHP2 | 5.0 | 200 | (83.0) | (83.0) |
| 1-RU^M^3 | rHP1 | 6.5 | 200 | <20.0 | 83.1 |
|  | rHP1 | 6.0 | 200 | <20.0 | 83.1 |
|  | rHP1 | 5.5 | 100 | 25.7 | 77.9 |
|  | rHP1 | 5.0 | 100 | 41.5 | 79.1 |
|  | dHP1 | 6.5 | 200 | 34.6 | 75.1 |
|  | dHP1 | 5.5 | 100 | (68.7) | (68.7) |
| 1-M12 | rHP1 | 5.5 | 100 | <20.0 | 78.2 |
|  | dHP1 | 6.5 | 200 | 37.1 | 75.4 |
|  | dHP1 | 5.5 | 100 | (69.5) | (69.5) |
| 1-MU^L^1 | rHP1 | 6.5 | 100 | <20.0 | 79.2 |
|  | rHP1 | 6.0 | 200 | <20.0 | 83.8 |
|  | rHP1 | 5.5 | 200 | <20.0 | 82.5 |
|  | rHP1 | 5.5 | 100 | <20.0 | 78.2 |
|  | dHP1 | 7.0 | 1000 | <20.0 | 80.6 |
|  | dHP1 | 6.8 | 1000 | <20.0 | 82.1 |
|  | dHP1 | 6.5 | 200 | 41.4 | 73.7 |
|  | dHP1 | 6.0 | 1000 | 53.4 | 81.2 |
|  | dHP1 | 6.0 | 200 | (73.5) | (73.5) |
| 1-MU^L^2 | rHP1 | 6.0 | 200 | <20.0 | 84.0 |
|  | rHP1 | 5.5 | 100 | <20.0 | 77.7 |
|  | dHP1 | 7.0 | 1000 | <20.0 | 80.7 |
|  | dHP1 | 6.8 | 1000 | 39.6 | 82.1 |
|  | dHP1 | 6.5 | 200 | (74.0) | (74.0) |
|  | dHP1 | 6.0 | 1000 | (81.5) | (81.5) |
|  | dHP1 | 6.0 | 200 | (73.5) | (73.5) |
| 1-MU^L^3 | rHP1 | 6.0 | 200 | <20.0 | 84.1 |
|  | rHP1 | 5.5 | 100 | <20.0 | 78.2 |
|  | dHP1 | 7.0 | 1000 | <20.0 | 80.7 |
|  | dHP1 | 6.8 | 1000 | 50.5 | 81.7 |
|  | dHP1 | 6.5 | 200 | (74.0) | (74.0) |
|  | dHP1 | 6.0 | 1000 | (81.2) | (81.2) |
|  | dHP1 | 6.0 | 200 | (73.5) | (73.5) |
| 1-Ms^2^U^M^1 | rHP1 | 5.5 | 100 | <20.0 | 78.6 |
|  | dHP1 | 7.0 | 1000 | <20.0 | 80.6 |
|  | dHP1 | 6.8 | 1000 | 36.9 | 82.1 |
|  | dHP1 | 6.5 | 200 | 42.9 | 74.0 |
|  | dHP1 | 6.0 | 1000 | (81.2) | (81.2) |
|  | dHP1 | 6.0 | 200 | (73.5) | (73.5) |
| 1-Ms^2^U^M^2 | rHP1 | 5.5 | 100 | <20.0 | 79.1 |
|  | dHP1 | 7.0 | 1000 | <20.0 | 80.7 |
|  | dHP1 | 6.8 | 1000 | 42.0 | 82.1 |
|  | dHP1 | 6.5 | 200 | (74.0) | (74.0) |
|  | dHP1 | 6.0 | 1000 | (81.5) | (81.5) |
|  | dHP1 | 6.0 | 200 | (73.5) | (73.5) |
| 1-Ms^2^U^M^3 | rHP1 | 5.5 | 100 | <20.0 | 78.5 |
|  | dHP1 | 7.0 | 1000 | <20.0 | 80.7 |
|  | dHP1 | 6.8 | 1000 | 50.0 | 81.7 |
|  | dHP1 | 6.5 | 200 | (74.0) | (74.0) |
|  | dHP1 | 6.0 | 1000 | (81.2) | (81.2) |
|  | dHP1 | 6.0 | 200 | (73.5) | (73.5) |
| 3-R12 | rHP3 | 7.0 | 200 | <20.0 | 91.9 |
|  | rHP3 | 6.5 | 200 | <20.0 | 91.2 |
|  | rHP3 | 6.0 | 1000 | <20.0 | >95.0 |
|  | rHP3 | 6.0 | 200 | 25.9 | 93.2 |
|  | rHP3 | 5.5 | 1000 | 25.2 | 93.5 |
|  | rHP3 | 5.5 | 200 | 43.6 | 93.5 |
|  | rHP3 | 5.5 | 100 | 53.0 | 93.7 |
| 3-Rs^2^U2 | rHP3 | 7.0 | 200 | <20.0 | 91.9 |
|  | rHP3 | 6.0 | 200 | 43.6 | 93.0 |
|  | rHP3 | 5.5 | 100 | 76.1 | 93.9 |
| 3-RU^L^2 | rHP3 | 7.0 | 200 | <20.0 | 92.1 |
|  | rHP3 | 6.5 | 200 | 24.7 | 93.4 |
|  | rHP3 | 6.0 | 200 | 43.8 | 93.4 |
|  | rHP3 | 5.5 | 200 | 68.2 | 93.0 |
|  | rHP3 | 5.5 | 100 | 74.2 | 93.0 |
| *^a^* The values shown in parentheses are for sequences with *T*_m1_ merging with *T*_m2_. The absorbance at 265 nm was recorded for 1-Rs^2^U3 binding to rHP1, rHP2, or dHP1. Experimental errors of *T*_m_ values are estimated as 1 °C. | | | | | |

**Table S2.** Melting temperatures and thermodynamic parameters of duplexes formed between various TFO strands (5 µM) and a purine strand (5′-AGAGAGAGAAAG-3′) (5 µM) in 200 mM NaCl at varying pH*^a^*

| TFO sequence | pH | *T*_m_ (°C) | | −Δ*H*^0^  (kcal/mol) | −Δ*S*^0^  (cal/mol·K) | −Δ*G*^0^_37_  (kcal/mol) |
| --- | --- | --- | --- | --- | --- | --- |
| 1-R12 | 7.0 | 54.0 | (54.3) | 89.6 | 247.8 | 12.69 |
|  | 6.0 | 54.3 | (55.0) | 93.7 | 259.6 | 13.13 |
|  | 5.5 | 51.9 | (53.0) | 87.3 | 241.8 | 12.30 |
| 1-RU^L^1 | 7.0 | 59.3 | (59.4) | 86.4 | 234.0 | 13.83 |
|  | 6.0 | 59.9 | (60.3) | 87.3 | 236.2 | 14.04 |
|  | 5.5 | 57.6 | (58.3) | 84.9 | 230.3 | 13.47 |
| 1-RU^L^2 | 7.0 | 65.7 | (66.4) | 93.2 | 248.9 | 16.01 |
|  | 6.0 | 67.9 | (68.0) | 96.5 | 258.0 | 16.51 |
|  | 5.5 | 63.2 | (65.1) | 96.2 | 258.4 | 16.02 |
| 1-RU^L^3 | 7.0 | 68.3 | (70.1) | 108.1 | 289.0 | 18.37 |
|  | 6.0 | 72.0 | (72.7) | 104.7 | 278.1 | 18.48 |
|  | 5.5 | 67.2 | (69.3) | 111.5 | 299.6 | 18.52 |
| 1-Rs^2^U1 | 7.0 | 57.6 | (58.6) | 82.7 | 223.9 | 13.27 |
|  | 6.0 | 57.4 | (58.2) | 79.1 | 213.1 | 13.05 |
|  | 5.5 | 56.4 | (57.0) | 80.4 | 217.7 | 12.88 |
| 1-Rs^2^U2 | 7.0 | 62.2 | (63.5) | 76.1 | 200.2 | 14.01 |
|  | 6.0 | 61.8 | (63.7) | 78.4 | 206.9 | 14.21 |
|  | 5.5 | 60.7 | (62.2) | 79.3 | 210.6 | 14.00 |
| 1-Ms^2^U^M^1 | 6.0 | 62.1 | (63.4) | 88.3 | 236.6 | 14.92 |
| 1-Ms^2^U^M^2 | 6.0 | 65.3 | (67.1) | 118.2 | 321.3 | 18.55 |
| 1-Ms^2^U^M^3 | 6.0 | 67.9 | (68.9) | 133.5 | 364.0 | 20.55 |
| 1-MU^L^1 | 6.0 | 67.8 | (68.4) | 111.9 | 320.7 | 18.03 |
| 1-MU^L^2 | 6.0 | 73.1 | (73.5) | 125.2 | 335.7 | 21.08 |
| 1-MU^L^3 | 6.0 | 75.5 | (76.5) | 135.2 | 360.1 | 23.17 |
| rHP1 | 6.5 | 84.1 | (84.7) | 115.3 | 322.2 | 15.35 |
|  | 6.0 | 84.0 | (84.5) | 114.6 | 320.3 | 15.22 |
|  | 5.5 | 83.5 | (84.1) | 110.4 | 309.1 | 14.55 |
| dHP1 | 6.5 | 73.7 | (73.9) | 89.5 | 257.8 | 9.51 |
|  | 6.0 | 73.5 | (73.7) | 92.2 | 265.8 | 9.77 |
|  | 5.5 | 74.4 | (74.6) | 88.7 | 255.1 | 9.59 |
| rHP2 | 6.0 | 83.8 | (84.5) | 114.1 | 319.1 | 15.15 |
|  | 5.5 | 83.8 | (84.7) | 117.1 | 327.1 | 15.61 |
| *^a^T*_m_ values shown in parentheses and thermodynamic parameters for duplex formation were obtained by fitting to a two-state model with the MeltWin program (<http://www.meltwin3.com>) (41). Thermal melting results for RS3 are not listed here due to the fact that the thermal melting transitions are non-two-state (see Fig. S6). The bottom three sequences are for RNA, DNA, and mutated RNA hairpins (see Fig. 2). Experimental errors of *T*_m_ values are estimated as 1 °C. Experimental errors of Δ*H*^0^, Δ*S*^0^, Δ*G*^0^_37_ (free energy at 37 °C) are estimated to as 12%, 13.5%, and 4%, respectively (59). | | | | | | |

**Table S3.** Melting temperatures and thermodynamic parameters of RNA hairpin (rHP1, Fig. 2A) in 200 mM NaCl at pH 6.0 in varying concentrations*^a^*

| Concentration (µM) | *T*_m_ (°C) | $-$Δ*H*^0^  (kcal/mol) | $-$Δ*S*^0^  (cal/mol·K) | $-$Δ*G*^0^_37_ (kcal/mol) |
| --- | --- | --- | --- | --- |
| 54.3 | 83.6 | 116.2 | 325.7 | 15.16 |
| 31.8 | 83.8 | 118.4 | 331.7 | 15.54 |
| 18.2 | 83.2 | 117.2 | 328.8 | 15.19 |
| 11.1 | 83.6 | 117.1 | 328.1 | 15.29 |
| 7.1 | 83.9 | 126.1 | 353.3 | 16.55 |
| 4.6 | 84.1 | 126.4 | 353.8 | 16.67 |
| 2.3 | 83.6 | 116.9 | 327.7 | 15.26 |
| 1.5 | 83.1 | 121.0 | 339.7 | 15.65 |
| 1.0 | 82.9 | 122.5 | 344.2 | 15.78 |

*^a^T*_m_ values and thermodynamic parameters were obtained by fitting to a two-state model with the MeltWin program (<http://www.meltwin3.com>) (41). Experimental errors of *T*_m_ values are estimated as 1 °C. Experimental errors of Δ*H*^0^, Δ*S*^0^, Δ*G*^0^_37_ (free energy at 37 °C) are estimated to as 12%, 13.5%, and 4%, respectively (59).

**
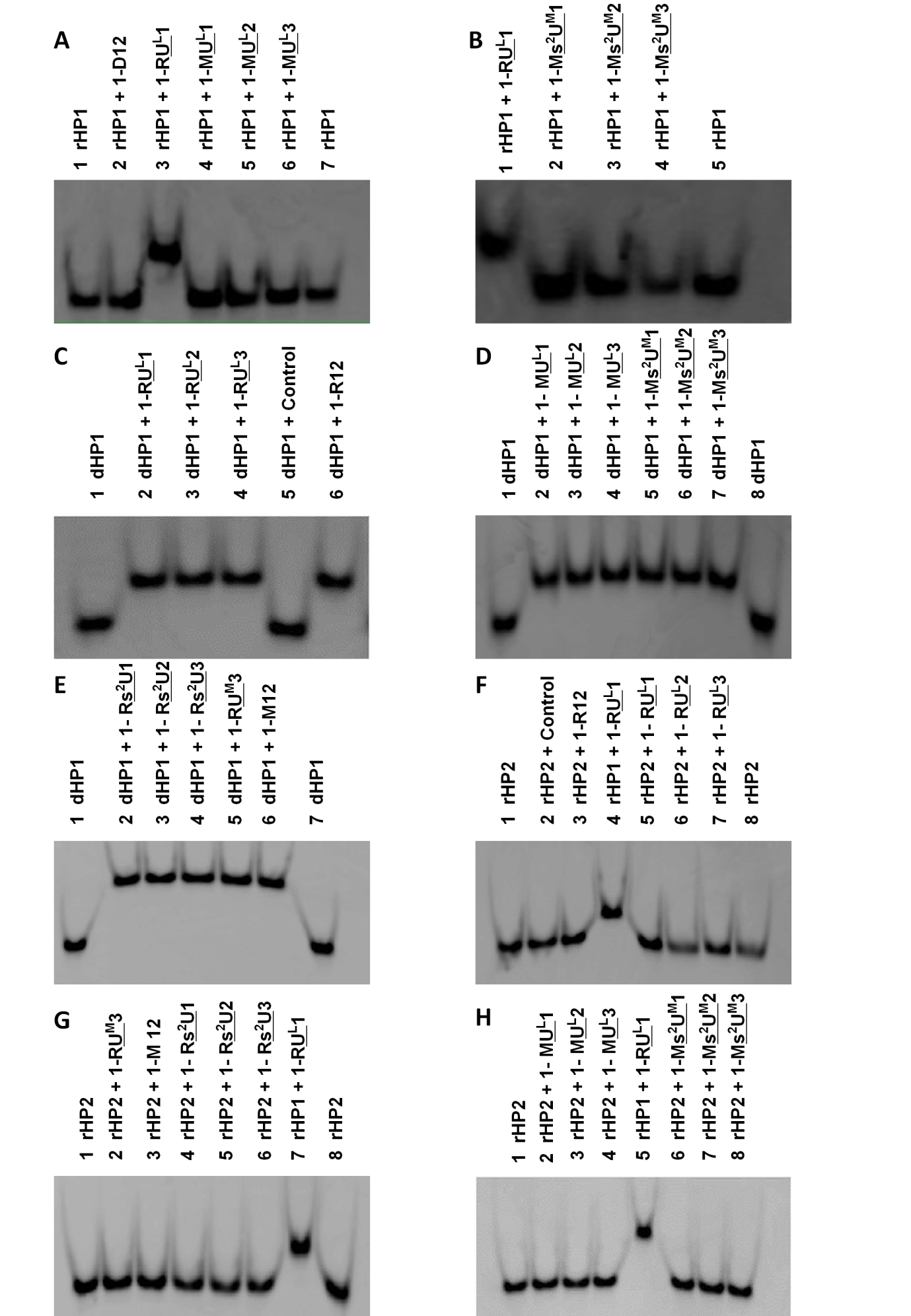
**

**Figure S1.** Native polyacrylamide gel for RNA hairpin 1 (rHP1, Fig 2A), mutated RNA hairpin 2 (rHP2, Fig 2C), DNA hairpin 1 (dHP1, Fig 2E), and triplexes. (**A**) TFOs 1-D12, 1-MU^L^1, 1-MU^L^2, and 1-MU^L^3 do not form triplexes with rHP1. (**B**) TFOs 1-Ms^2^U^M^1, 1-Ms^2^U^M^2, and 1-Ms^2^U^M^3 do not form triplexes with rHP1. (**C**) TFOs 1-R12, 1-RU^L^1, 1-RU^L^2, and 1-RU^L^3 form triplexes with dHP1. (**D**) TFOs 1-MU^L^1, 1-MU^L^2, 1-MU^L^3, 1-Ms^2^U^M^1, 1-Ms^2^U^M^2, and 1-Ms^2^U^M^3 form triplexes with dHP1. (**E**) TFOs 1-Rs^2^U1, 1-Rs^2^U2, 1-Rs^2^U3, 1-RU^M^3 and 1-M12 form triplexes with dHP1. (**F**) TFOs 1-R12, 1-RU^L^1, 1-RU^L^2, and 1-RU^L^3 do not form triplexes with rHP2. (**G**) TFOs 1-RU^M^3, 1-M12, 1-Rs^2^U1, 1-Rs^2^U2, and 1-Rs^2^U3 do not form triplexes with rHP2. (**H**) TFOs 1-MU^L^1, 1-MU^L^2, 1-MU^L^3, 1-Ms^2^U^M^1, 1-Ms^2^U^M^2, and 1-Ms^2^U^M^3 do not form triplexes with rHP2.





**Figure S2.** Hypothetical structures formed involving TFO strands. A representative TFO strand is show in gray with the residues to be modified shown in black. Note that our results suggest that structures shown in panels (A, B, and D) do not form. (**A**) Parallel duplex structure formed by a modified TFO binding to the RNA hairpin sequence, rHP1 (Fig. 2A). (**B**) Antiparallel duplex structure formed by a TFO binding to rHP1. (**C**) 9-bp antiparallel duplex structure formed between a 12-nt purine strand and a TFO. The 12-nt purine strand has the same sequence as the purine segment of rHP1. (**D**) 12-bp parallel duplex structure formed between the 12-nt purine strand and a TFO.


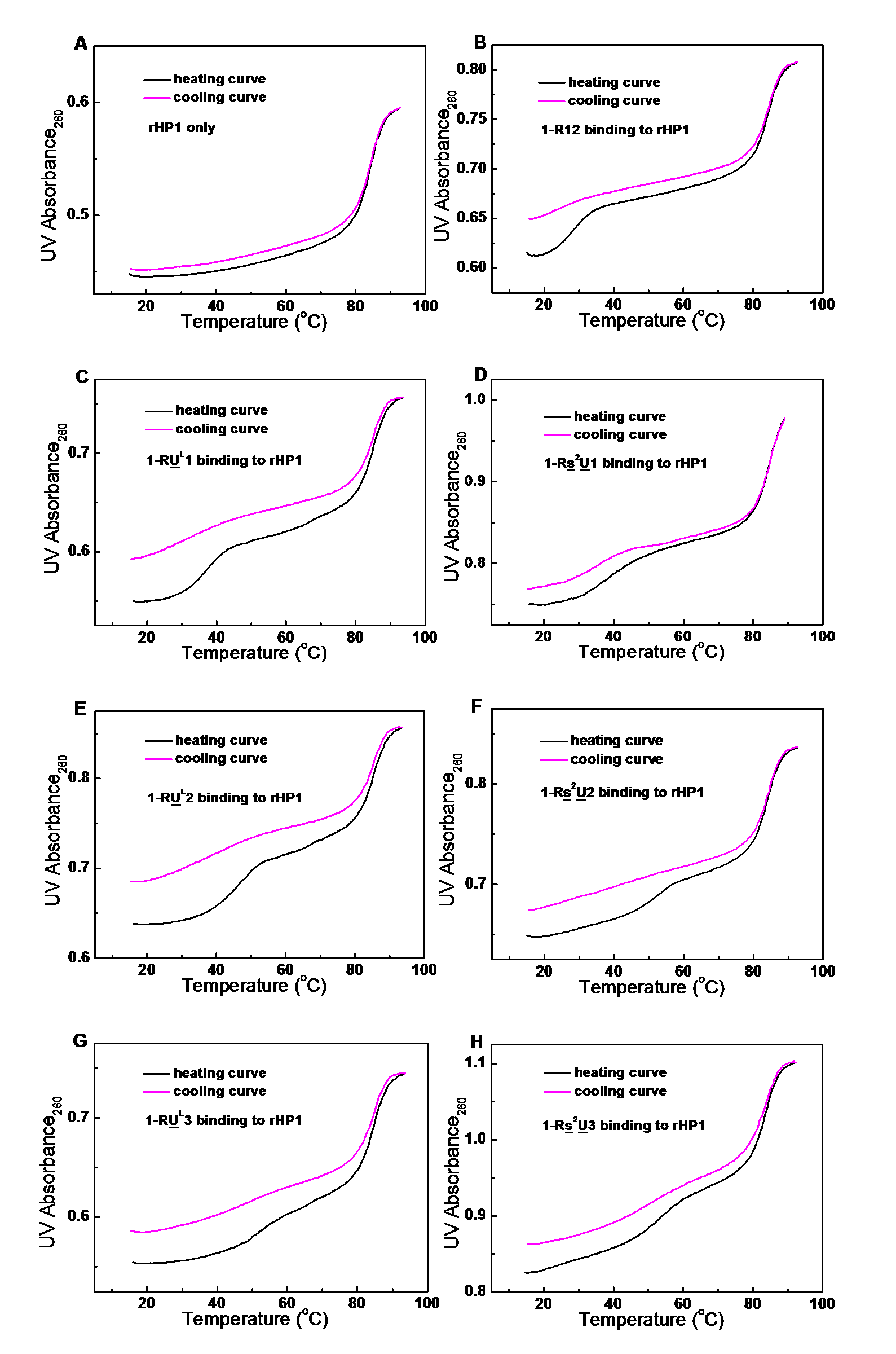


**Figure S3.** UV-absorbance-detected thermal melting curves (heating and cooling) of various TFOs binding to the RNA hairpin (rHP1) in 200 mM NaCl at pH 6.0. It reveals that *T*_m1_ in the heating curve is higher than that in the cooling curve, indicating a hysteresis for triplex to hairpin transition. *T*_m2_ is almost the same for heating and cooling curves, indicating no hysteresis for rHP1 to single strand transition.


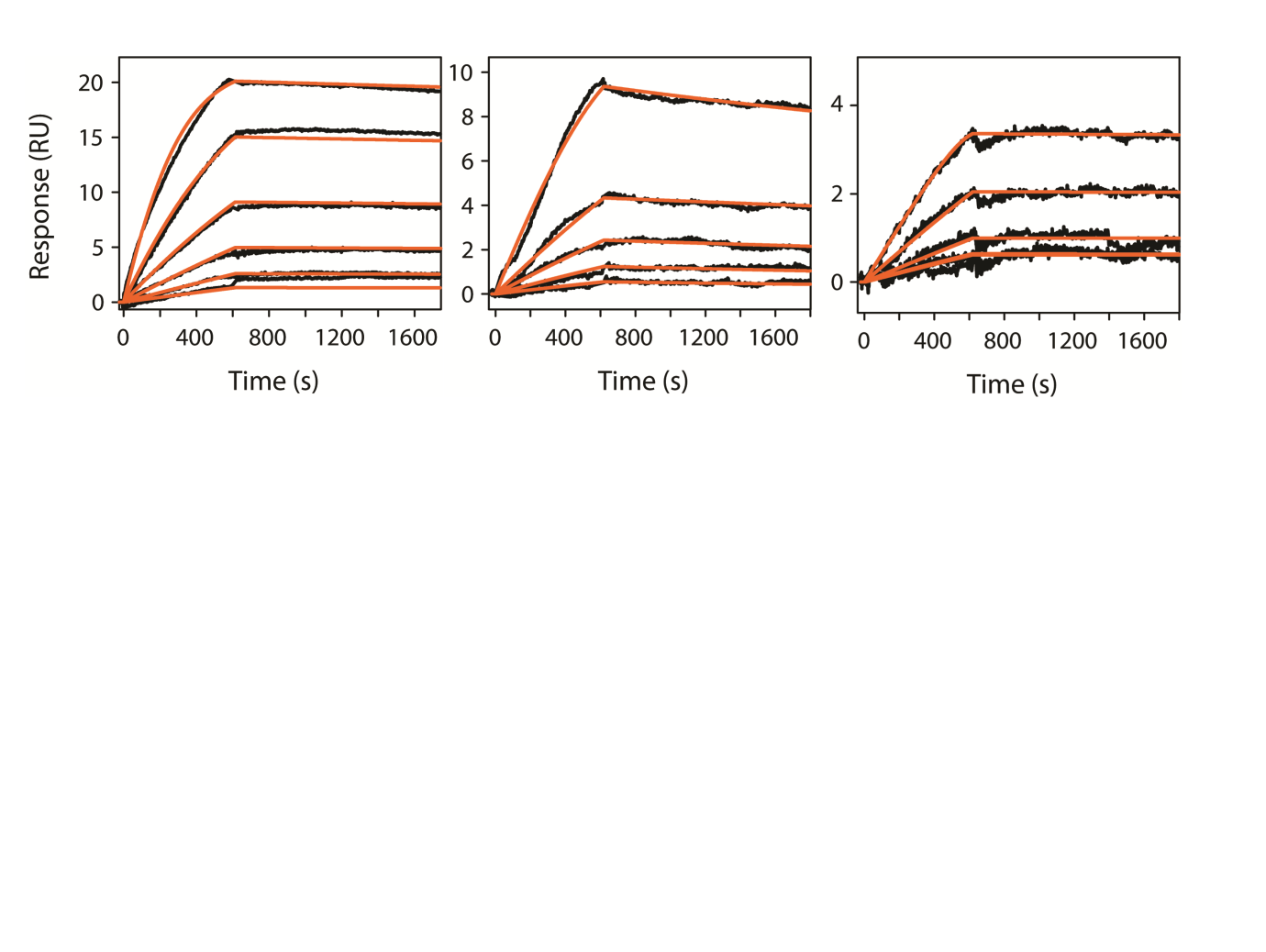


| **TFO** | ***k*_a_ (M^-1^s^-1^)** | ***k*_d_ (**$\boldsymbol{\times}$**10^-5^ s^-1^)** | ***K*_D_ (**$\boldsymbol{\times}$**10^-9^ M)** |
| --- | --- | --- | --- |
| 3-R12 (n=6) | 270 ± 23 | 2.5 ± 0.3 | 93 ± 13 |
| 3-Rs^2^U2 (n=5) | 330 ± 2 | 19.0 ± 5.3 | 570 ± 162 |
| 3-RU^L^2 (n=4) | 1360 ± 333 | 0.95 ± 0.01 | 7.0 ± 6.9 |

**Figure S4.** SPR measurements of the interactions between various TFOs and RNA hairpin, rHP3. 82 nM, 247 nM, 741 nM, 2.2 μM, 6.7 μM, and 20 μM of TFOs were serially injected, and the highest response is 20 μM. The binding dissociation constants, *K*_D_ was calculated based on *K*_D_ = *k*_d_/ *k*_a_. We note that both dissociation and association rates are slow, which makes it difficult to measure kinetics accurately.


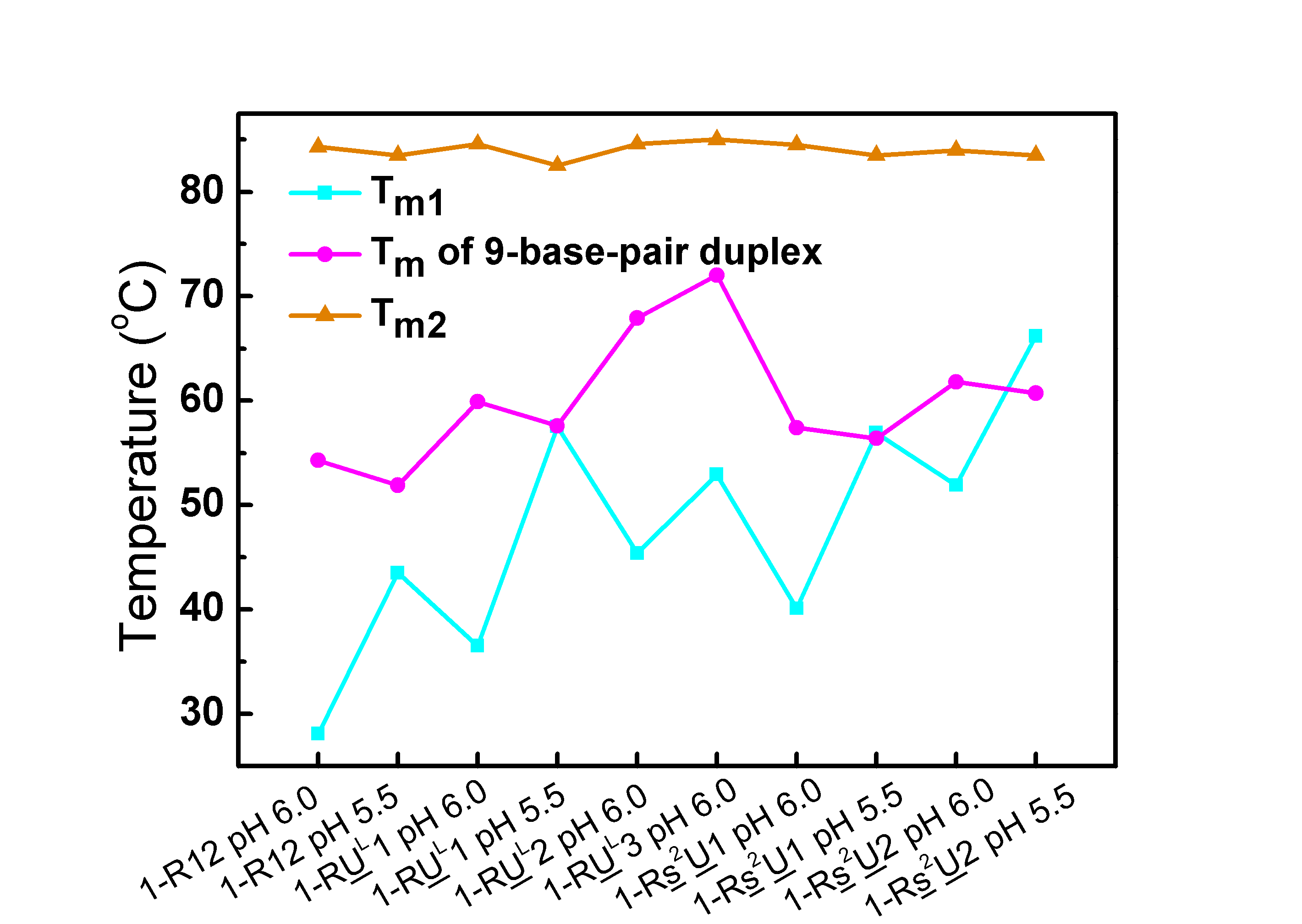


**Figure S5.** Comparison of melting temperatures of triplexes (Fig. 2B, *T*_m1_), RNA hairpin rHP1 (Fig. 2A, *T*_m2_), and 9-bp duplexes (Fig. S2C, *T*_m_) in 200 mM NaCl at varying pH.


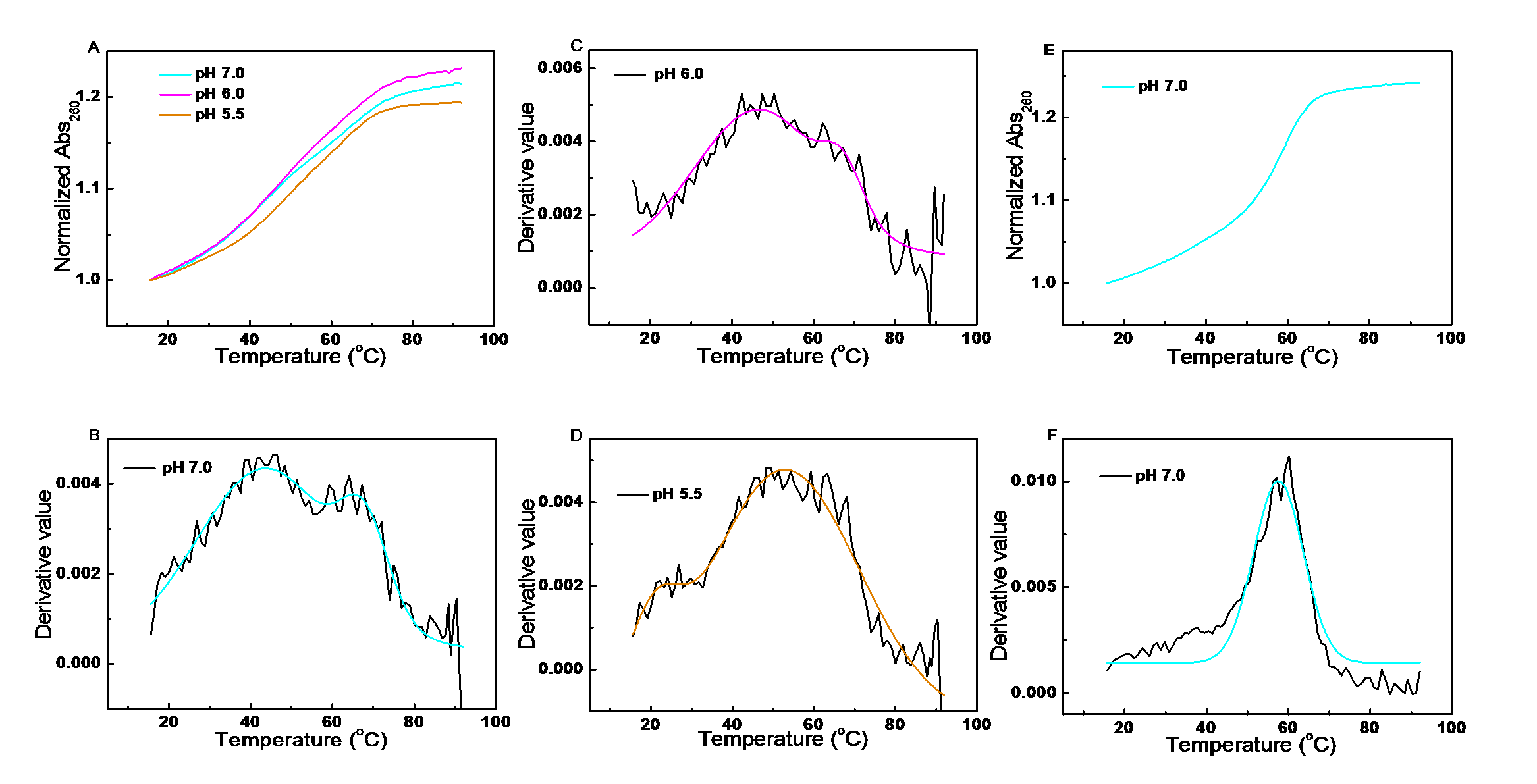


**Figure S6.** (**A**) UV-absorbance-detected thermal melting curves of duplexes formed between the TFO RS3 and the purine strand (5′-AGAGAGAGAAAG-3′) in 200 mM NaCl at pH 7.0 (cyan), 6.0 (magenta), and 5.5 (orange). (**B-D**) First derivatives of thermal melting curves in panel (A) reveal that the thermal melting transitions are non-two-state. The Gaussian fits of the first derivative curves are in the same color codes as panel (A). (**E**) Thermal melting curve of the TFO 1-Rs^2^U1 binding to the purine strand (5′-AGAGAGAGAAAG-3′) in 200 mM NaCl at pH 7.0. (**F**) First derivative of the thermal melting curve in panel (E) suggests that it is a two-state transition.

**REFERENCES**

41. McDowell, J. A., and Turner, D. H. (1996) Investigation of the structural basis for thermodynamic stabilities of tandem GU mismatches: solution structure of (rGAGGUCUC)_2_ by two-dimensional NMR and simulated annealing, *Biochemistry* **35**, 14077-14089.

59. Xia,T., SantaLucia,J.,Jr., Burkard,M.E., Kierzek,R., Schroeder,S.J., Jiao,X., Cox,C. and Turner,D.H. (1998) Thermodynamic parameters for an expanded nearest-neighbor model for formation of RNA duplexes with Watson-Crick base pairs. *Biochemistry*, **37**, 14719-14735.
